# Repeated pulmonary dosing of β-glucan-chitosan-PLGA nanoparticles controls *Mycobacterium tuberculosis* in mice

**DOI:** 10.1128/aac.01480-25

**Published:** 2026-01-14

**Authors:** Hilliard L. Kutscher, Maria Tamblin, Evon Smith, Arnav Shah, Patrick O. Kenney, Jessica L. Reynolds

**Affiliations:** 1 Division of Allergy, Immunology, and Rheumatology Department of Medicine, Clinical and Translational Research Center, The State University of New York at Buffalo, Buffalo, New York, USA; 2 Institute for Lasers, Photonics and Biophotonics, The State University of New York at Buffalo, Buffalo, New York, USA; 3 Department of Anesthesiology, The State University of New York at Buffalo, Buffalo, New York, USA; 4 Adult and Pediatric Infectious Disease, The State University of New York at Buffalo, Buffalo, New York, USA; City St George's University of London, London, United Kingdom

**Keywords:** *Mycobacterium tuberculosis*, rifampin, nanoparticles, PLGA, beta glucan, immune profiling

## Abstract

To address limitations in tuberculosis (TB) therapy, we developed an inhalable, immunomodulating, biocompatible nanoparticle system (β-C-P) encapsulating rifampin that targets alveolar macrophage. The nanoparticle consists of a poly(lactic-co-glycolic acid) (PLGA) core, a chitosan coating, and a surface functionalized with 1,3-β-glucan for enhanced macrophage uptake and immunomodulation. We evaluated the safety, immunological effects, and efficacy of rifampin-loaded β-C-P nanoparticles delivered via oropharyngeal aspiration (OPA) in healthy mice and in a low-dose *Mycobacterium tuberculosis (Mtb*) BALB/c model treated weekly for 4 weeks, as well as in a low-dose *Mtb* C3HeB/FeJ model treated weekly for 8 weeks. In healthy mice, cell pellets isolated by bronchoalveolar lavage (BAL) showed higher pulmonary exposure (AUC) of rifampin with 20% β-C-P nanoparticles versus 5% β-C-P nanoparticles, while no rifampin was detected in the oral rifampin group. Flow cytometry revealed no significant changes in lung immune cell populations except for a transient neutrophil increase at day 21 in the 5% β-C-P group. In the *Mtb* BALB/c mouse model, weekly OPA administration of 5%, 10%, and 20% β-C-P nanoparticles significantly reduced lung CFU by 0.5–1.11 log_10_, comparable to daily oral rifampin. In the *Mtb* C3HeB/FeJ (Kramnik) mouse model, weekly OPA administration of 10% and 20% β-C-P nanoparticles significantly reduced lung CFU, comparable to daily oral rifampin. Collectively, these findings demonstrate that weekly pulmonary nanoparticle delivery of rifampin-loaded β-C-P nanoparticles achieves sustained rifampin exposure and therapeutic efficacy comparable to daily dosing, without pulmonary toxicity or systemic immune activation. This supports the potential of long-acting inhalable formulations for simplified TB therapy.

## INTRODUCTION

Tuberculosis (TB), caused by *Mycobacterium tuberculosis* (*Mtb*), remains a leading global health threat. In 2023, TB was responsible for an estimated 1.25 million deaths and once again became the single most-deadly infectious disease worldwide (1). Despite being a curable disease, TB mortality rates remain high due to multiple factors. The standard TB treatment regimen involves multiple antibiotics administered over a prolonged course of 3 to 9 months, depending on drug sensitivity and resistance (2). While effective, this extended timeline, combined with the systemic toxicity of many first-line drugs, contributes to poor patient adherence, treatment failure, and the emergence of drug-resistant TB (MDR-TB) (3, 4).

Rifampin, a cornerstone of TB therapy, has potent sterilizing activity but is often associated with hepatotoxicity and subtherapeutic lung concentrations when administered orally (5–7). These pharmacokinetic limitations underscore the need for improved drug delivery systems that increase therapeutic concentrations at the infection site while reducing systemic toxicity. Inhalable drug delivery systems offer a promising alternative by enabling direct delivery of therapeutics to the lungs, the primary site of infection, thereby enhancing local drug concentrations, avoiding first-pass metabolism, and reducing off-target side effects (8–10). Nanoparticle-based inhalable systems further enable precision delivery to alveolar macrophages, which serve as intracellular reservoirs for *Mtb* (11, 12). However, many existing nanoparticle formulations lack cell-specific targeting, exhibit poor uptake by host immune cells, lack immune stimulation, or fail to sustain drug release at therapeutic levels. To address these challenges, we developed a macrophage-targeting, inhalable nanoparticle drug delivery system encapsulating rifampin. The formulation (β-C-P) consists of a poly(lactic-co-glycolic acid) (PLGA) core and a chitosan shell, functionalized with 1,3-β-glucan. β-Glucan was selected not only for its ability to enhance macrophage uptake via dectin-1 receptors but also for its immunostimulatory effects, which can support host defense mechanisms against *Mtb* (11–14). This system is designed to improve pulmonary drug retention, increase intracellular antibiotic concentration, and reduce systemic toxicity. Previous studies from our group have evaluated the β-C-P nanoparticle system using both *in vitro* and *in vivo* pharmacokinetic and pharmacodynamic analyses (12–14). *In vitro*, β-C-P nanoparticles demonstrated immunostimulatory activity in macrophages, as evidenced by increased production of pro-inflammatory cytokines (TNF-⍺, IL-6, and IL-1β) and reactive oxygen species (11). These findings supported the optimization of β-glucan as a surface ligand on the nanoparticles to enhance macrophage targeting. Additionally, treatment of *Mtb*-infected macrophages with β-C-P nanoparticles *in vitro* resulted in a significant reduction in intracellular bacterial burden (14). Our previous *in vivo* studies assessed the pharmacokinetics and safety of a single inhaled dose of β-C-P nanoparticles in mice. Compared to oral rifampin, the oropharyngeal aspiration (OPA)-administered β-C-P formulation led to higher rifampin concentrations in the lungs, with drug levels sustained for up to 7 days following administration. Importantly, the single dose was well tolerated, as confirmed by cytokine profiling, immune cell characterization, and histological analysis (12).

Unlike previous inhalable systems (15), our formulation is designed to specifically target and stimulate alveolar macrophages while sustaining therapeutic rifampin concentrations in the lungs and minimizing systemic exposure. This design raises the possibility of less frequent dosing, which could dramatically improve patient adherence, especially in resource-limited settings where directly observed therapy is difficult to maintain. In this study, we evaluated the long-term pharmacokinetics and efficacy of β-C-P nanoparticles and compared once-weekly inhalable β-C-P nanoparticle treatment with daily oral rifampin using two validated aerosol low-dose *Mtb* models. The first being the BALB/c mouse model, which recapitulates key features of human TB pathology (5–11). This low-dose aerosol infection model demonstrates a predictable disease course and progression, with pulmonary infection by *Mtb* resulting in homogeneous, multifocal lung lesions that closely mimic human pathology (8, 16–21). Additionally, immune biomarkers associated with TB progression and treatment response have been thoroughly characterized in this model (22, 23), enhancing its utility for immunological and pharmacological studies. It is widely accepted for pre-clinical evaluation of anti-TB therapeutics, particularly those delivered through the pulmonary route (8, 22, 24–28). The second model is the C3HeB/FeJ (Kramnik) mouse model. Unlike BALB/c mice, which develop primarily cellular lesions, the Kramnik model more closely mimics human TB pathology, especially in terms of granuloma formation, immune response, and susceptibility to chronic infection, making it a critical model for studying the disease (24, 29–33). Importantly, therapies that appear effective in BALB/c mice often fail to demonstrate efficacy in Kramnik mice, highlighting the necessity of testing novel interventions in this model (24, 33, 34). By delivering β-C-P nanoparticles through inhalation in both BALB/c and Kramnik models, we aimed to improve macrophage-targeted delivery, enhance drug penetration into necrotic granulomas, and achieve superior therapeutic outcomes. We hypothesized that once-weekly inhalation of β-C-P nanoparticles would maintain therapeutic rifampin concentrations in the lungs and enhance antibacterial efficacy compared to daily oral rifampin therapy. Ultimately, this strategy could inform the development of next-generation TB therapies that are shorter in duration, safer, and more effective.

## MATERIALS AND METHODS

### Materials

PLGA (50:50 ester-terminated 24-38 kDa MW), chitosan oligosaccharide lactate (C, 5 kDa MW), 1,3-β-glucan (β-glucan), polyvinyl alcohol (Molwiol 4-88, 31 kDa MW), and rifapentine were purchased from MilliporeSigma (St. Louis, MO). Methylene chloride (ACS reagent grade, ≥99.5%) and acetonitrile were purchased from Fisher Scientific. Rifampin (>98%) was purchased from TCI America (Portland, OR). All materials were used as received. Kollisolv PYR was a generous gift from BASF Corp. (Florham Park, NJ).

### Synthesis of β-glucan-chitosan-PLGA nanoparticles

Rifampin-loaded β-glucan-chitosan-PLGA (β-C-P) nanoparticles were prepared via a water/oil/water emulsion, followed by solvent evaporation as previously described (12). The resulting nanoparticles are referred to as β-C-P nanoparticles (Table 1). The content of surface β-glucan bound by electrostatic binding was determined using a Glucatell assay kit (Associates of Cape Cod, East Falmouth, MA), as previously described (12, 35).

**TABLE 1:** Description of treatments and nanoparticle concentrations of PLGA, β-glucan, and rifampin in a single bolus dose, depending on the percentage of nanoparticle solids administered by OPA or oral gavage^*a*^

Label	% Nanoparticle solids (wt/vol)	PLGA (mg/kg)	Surface β-glucan (ng/mg PLGA)	Rifampin (mg/kg)	Dose route	Dose frequency
Control (no treatment)	N/A	N/A	N/A	N/A	N/A	N/A
PBS (vehicle control)	N/A	N/A	N/A	N/A	OPA	Weekly
Rifampin alone	N/A	N/A	N/A	4.33	Gavage	Daily (5/7)
20% β-C-P	20%	333	25	4.33	OPA	Weekly
10% β-C-P	10%	166.5	25	2.17	OPA	Weekly
5% β-C-P	5%	83.25	25	1.08	OPA	Weekly

^*a*^ 20% solids = 200 mg/mL PLGA = 10 mg PLGA/50 µL dose = 333 mg PLGA/kg body weight = 4.33 mg rifampin/kg; N/A = not applicable.

### Nanoparticle characterization

The hydrodynamic diameter and surface charge of the resulting β-C-P nanoparticles were determined using dynamic light scattering (DLS) and zeta potential analysis performed on an SZ-100 Nanopartica (Horiba Instruments, Inc., Irvine, CA). To confirm size, scanning electron microscopy (SEM) was performed as previously described (12).

### Rifampin concentration determination

The amount of rifampin in the β-C-P nanoparticles was determined using a Nanodrop 2000 at 338 nm, blanked against acetonitrile. Concentrations were determined using a quadratic standard curve, from 2.5 to 250 µg/mL (*R*^2^=0.9919). Nanoparticles were dissolved 1:40 in acetonitrile and bath-sonicated (12).

### Healthy mice used for non-infectious studies

Four- to 6-week-old male CD1 mice (Charles River Laboratories, Wilmington, MA) were housed in microisolator cages and maintained with sterile bedding, water, mouse chow, and a standard light and dark cycle. Drinking water and food were freely available.

### OPA and oral gavage

For OPA, isoflurane-anesthetized mice were administered a 50 µL bolus dose of rifampin-loaded β-C-P nanoparticles or vehicle to the back of the throat, and the mouse inhaled the liquid, resulting in delivery to the lungs, as previously described (12, 36). For oral gavage, mice were administered a 50 µL bolus dose of rifampin (2.5 mg/mL in 2.5% [vol/vol] Kollisolv PYR in PBS) using a 20 Ga feeding tube (Instech, Plymouth Meeting, PA), 5 out of 7 days (12). The dose of nanoparticles or rifampin is shown in Table 1.

### Bronchoalveolar lavage (BAL) collection

BAL was performed, and cell pellets and supernatant were isolated as previously described (12, 36). Twenty microliters of acetonitrile was added, and samples were stored at -20°C until HPLC quantitation.

### Recovery of lungs

Lungs were dissected, weighed, and placed in a C-tube (Miltenyi Biotec, Gaithersburg, MD) containing 4 mL of Hanks’ Balanced Salt Solution on ice until further processing described below (12, 14).

### Rifampin concentration in cells recovered by BAL

Rifampin detection was performed on a Shimadzu LC20 series HPLC with an Xbridge Phenyl 3.5 µm 2.1 × 100 mm column (Waters Corp., Milford, MA) and detected by UV-VIS at 338 nm, as previously described (12).

### Pharmacokinetics and AUC

AUC was computed using the R package PK (37), as previously described (12).

### ELISA

Cytokines in BAL supernatants were analyzed using mouse DuoSet ELISA kits for TNF-α, IL-6, and IL-1β, according to the manufacturer’s instructions (DY410, DY406, and DY401; R&D Systems) and as previously described (12). Albumin concentration was analyzed from BAL supernatants using a Mouse Albumin ELISA Kit (ab108791; Abcam), as previously described (12). Serum collected from mice was analyzed for IgG concentrations using an ELISA kit (88-50400; Invitrogen).

### Lung disassociation and antibody staining

Lung tissue was dissociated using a gentleMACS Octo Dissociator with heaters (Miltenyi Biotec) at setting 37 mLDK, as previously described (12). Cells were stained using a master mix of six antibodies (Ly6C [PerCp-Cy5.5], CD45 [Alexa Fluor 700], CD5 [BV421], CD19 [BV650], CD11b [BV510], and CD11c [Alexa Fluor 488]; BioLegend, San Diego, CA), as previously described (12). Samples were analyzed on a BD FACS Celesta. The flow cytometer was compensated using fluorescence minus one for identification of positive populations for each channel used. Results were analyzed using FlowJo (v.10; BD Biosciences, Ashland, OR).

### *In vivo Mtb* studies

Eight-week-old male and female BALB/c mice (Envigo, Indianapolis, IN) were housed in an A-BSL-3 animal facility and maintained with sterile bedding, water, mouse chow, and exposed to a standard 12 h light and dark cycle. Six- to 8-week-old female C3HeB/FeJ mice (Jackson Laboratories, Bar Harbor, ME) were housed in an A-BSL-3 animal facility with sterile bedding, water, and mouse chow and exposed to a standard 12 h light and dark cycle.

### *Mtb* growth and aerosol exposure

For both models, *Mtb* Erdman strain (Strain Erdman K01 [TMC 107] NR-15404; BEI resources) grown in Middlebrook 7H9 broth with ADC growth supplement and 0.05% Tween 80 was used for aerosol infections of mice. Mice were exposed to a low-dose aerosol infection using an AERO3G Whole Body Inhalation System (Biaera Technologies, Hagerstown, MD), resulting in an average of 50 to 100 *Mtb* bacilli in the lungs of each mouse (24, 29–33).

### BALB/c-*Mtb* Model

**Fig 1:**
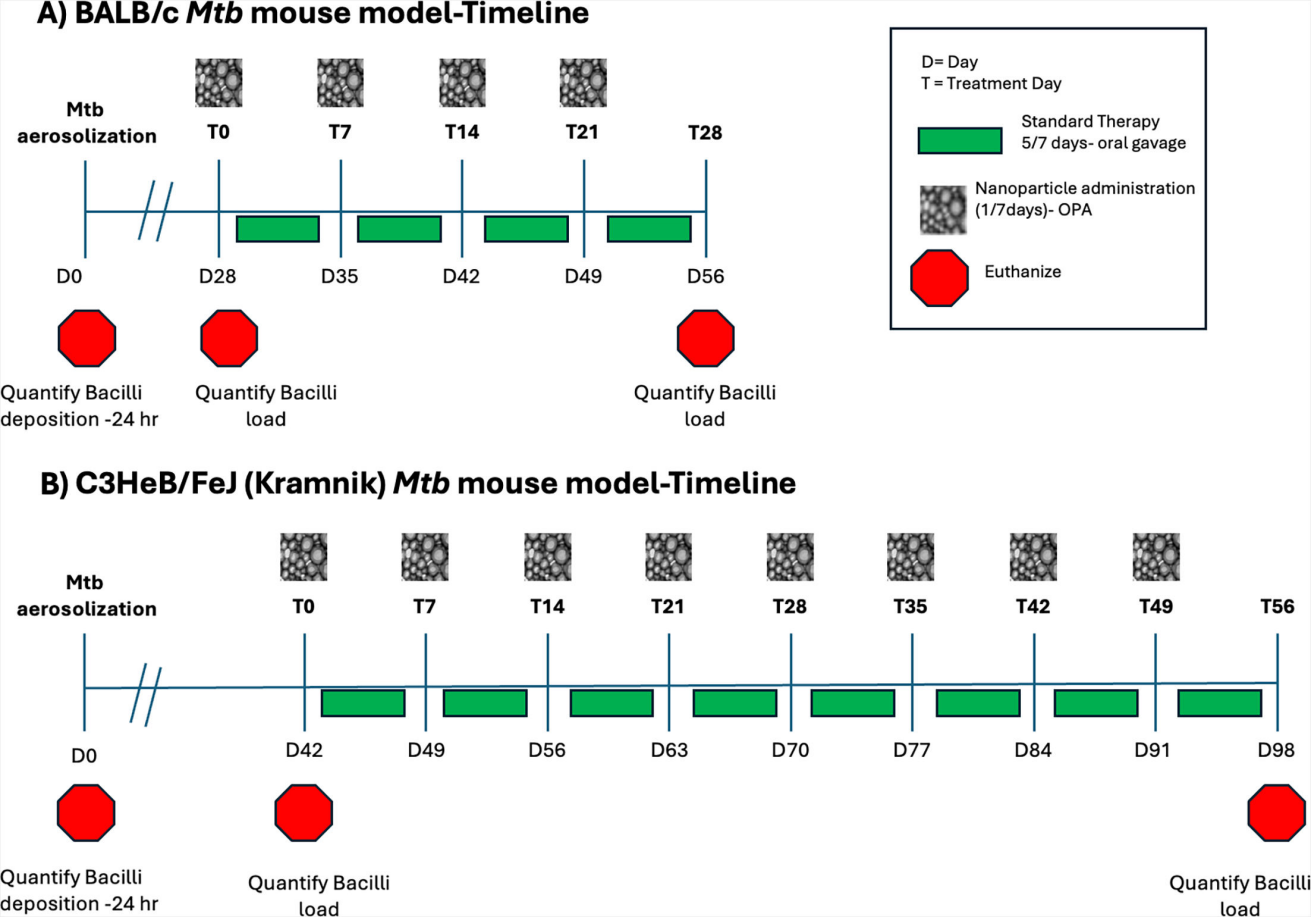
*Mtb* infection model timeline. (**A**) BALB/c model. (**B**) C3HeB/Fej Kramnik model.

On day 1 post-infection (24 h), five mice were euthanized to determine the number of CFU implanted in the lungs (8, 22, 26). Mice were observed weekly and weighed once a week on each cage change day. At 28 days post-infection, five mice were euthanized to determine starting bacterial load (8, 22, 26). Starting on day 28, mice were monitored 5 out of 7 days, and clinical symptoms, including appearance, level of consciousness, activity, response to stimulus, eye condition, respiration rate, and respiration quality, were scored from 0 to 4 (38, 39). All treatments were initiated at day 28 post-TB infection, which is referred to as treatment day 0 (T0). Nanoparticles (dosed every 7 days) were given on T0, T7, T14, and T21. Rifampin (Table 1) was dosed by oral gavage 5 out of 7 days each week (Fig. 1A, Timeline). Mice were euthanized on T28.

### C3HeB/FeJ (Kramnik)-*Mtb* model

Day 1 post-infection (24 h), five mice were euthanized to determine the CFU in the lungs. Daily observation of mice was performed to assess clinical symptoms such as appearance, level of consciousness, activity, response to stimulus, eye condition, respiration rate, and respiration quality (38). Mice were weighed once a week during cage change. On days 21–42, mice were observed twice daily based on the increased potential mortality in this mouse model (24, 32). On day 42 post-infection, 5 mice were euthanized to determine the starting bacterial load. All treatments were initiated on day 42 post-TB infection, which is referred to as treatment day 0 (T0). Nanoparticles were administered every 7 days (T0, T7, T21, T28, T35, T42, T49) (Fig. 1B, Timeline) by OPA, and oral rifampin was administered by gavage 5 of 7 days. Mice were euthanized on T56 (day 98).

### TB-infected lungs

Whole lungs from infected mice were aseptically removed, weighed, and disrupted in 4 mL of ice-cold PBS using a Precellys Evolution homogenizer (Bertin Corporation, Rockville, MD). Serial dilutions of homogenized whole lungs in PBS were plated on 7H11 agar plates supplemented with cycloheximide (0.1 mg/mL), carbenicillin (0.5 mg/mL), and 0.4% activated charcoal. Activated charcoal was used to prevent drug carryover (8, 22, 24, 26, 33). Colonies were enumerated after a minimum of 28 days of incubation at 37°C. All plates were incubated up to 8 weeks for complete detection of all colonies. The viable bacterial counts of whole organs were calculated and converted to logarithms (8, 22, 24, 26, 33).

### Spleen

The spleens from infected mice were aseptically removed, weighed, and disrupted in 2 mL of ice-cold PBS using a Precellys Evolution homogenizer. Serial dilutions of homogenized spleen in PBS were plated on 7H11 agar plates. Colonies were enumerated after a minimum of 28 days of incubation at 37°C. All plates were incubated up to 8 weeks for complete detection of all colonies. The viable bacterial counts of whole spleens were calculated and converted to logarithms (8, 22, 24, 26, 33).

### Statistical analysis

Analysis was done by a one-way or two-way analysis of variance (ANOVA), followed by Tukey’s or Dunnett’s correction for multiple comparisons, as indicated in the figure legends. All analyses were performed using Prism (v.10.4; GraphPad Software, San Diego, CA).

## RESULTS

### Characterization of β-C-P nanoparticles

**Fig 2:**
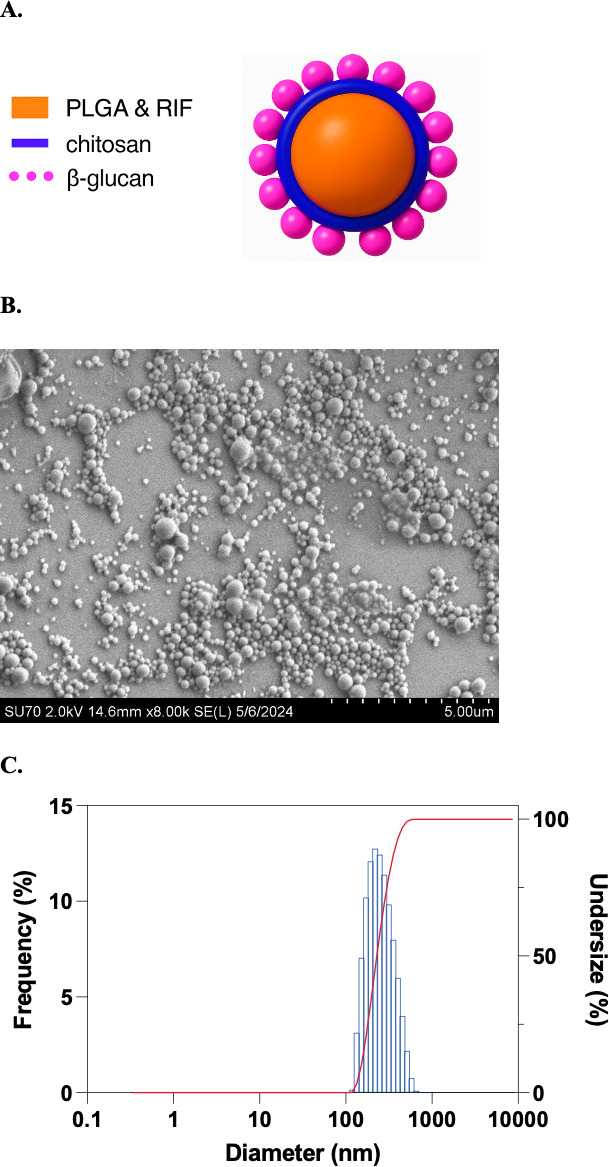
β-C-P nanoparticle characterization. Schematic (**A**), representative image (**B**) of β-C-P nanoparticles using SEM demonstrating morphology, and (**C**) DLS measurement confirming size. PLGA, poly(lactic-co-glycolic acid); Rif, rifampin.

Rifampin-loaded β-C-P nanoparticles were synthesized using a water-in-oil-in-water (w/o/w) emulsion-solvent evaporation method, as previously described and extensively characterized (11–13) (Fig. 2A). The core nanoparticles (C-P) were prepared by sonication, followed by overnight stirring to allow for polymer hardening. Chitosan (C) coating was applied to the PLGA surface during nanoparticle formation to enhance nanoparticle stability and impart a positive surface charge (zeta potential: 22.8 ± 6.4 mV). The positive surface charge enhances cellular uptake (13), likely due to the electrostatic interaction between the positively charged particle and the negatively charged cell surface. After passing through a 40 µm cell strainer and multiple centrifugation steps to remove free rifampin and excess surfactant, the resulting C-P nanoparticles were resuspended in PBS and surface-functionalized with 1,3-β-glucan. The β-glucan serves both as a targeting ligand and as an immunostimulatory agent. Final β-C-P nanoparticles were spherical and uniform in morphology (Fig. 2B), with an average hydrodynamic diameter of 237 ± 23.7 nm (PDI: 0.166), as measured by DLS (Fig. 2C) and a zeta potential of 3.3 ± 3.3 mV, consistent with previously reported values (14).

### Pharmacokinetics of inhaled β-C-P nanoparticles

**Fig 3:**
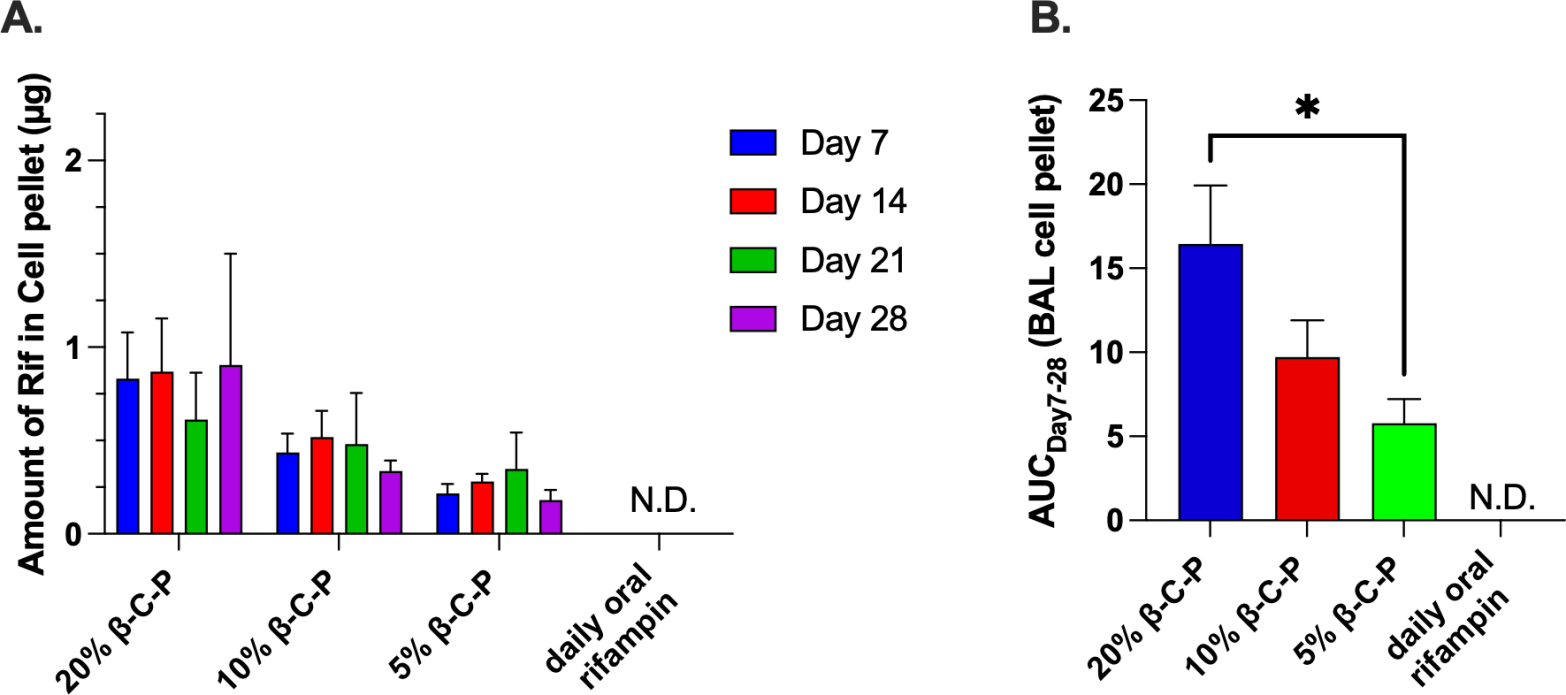
Analysis of rifampin in the BAL cell pellet following repeated inhalation of β-C-P nanoparticles. (**A**) Amount of rifampin in the cell pellet collected from the lungs. (**B**) Area under the curve (AUC) analysis. Statistical analysis was done by a two-way ANOVA followed by Tukey’s multiple comparisons test (**A**) and a one-way ANOVA with Tukey’s multiple comparisons test (**B**). Data shown represent the mean ± SEM (*n* = 4). *P ≤ 0.05, N.D., not detectable.

Given that alveolar macrophages are one of the primary host cells for *Mtb* in the lung (40–42), we delivered β-C-P nanoparticles via OPA to enable direct targeting of these immune cells. This inhalation-based route mimics aerosol administration and bypasses gastrointestinal metabolism (9, 10). Building on our previous findings demonstrating prolonged lung exposure of rifampin for up to 7 days after a single OPA dose (12), likely due to the controlled release of rifampin (13) and the sustained presence of the β-C-P nanoparticles in the lung (12), we extended our investigation to a 4 week treatment regimen to evaluate pharmacokinetics, safety, and efficacy in a murine model. Drug exposure in alveolar macrophages was quantified by measuring rifampin concentration in the BAL cell pellet collected on days 7, 14, 21, and 28, 1 week post last dose. Rifampin levels remained below the limit of detection in BAL following oral delivery of rifampin. In contrast, mice treated with β-C-P nanoparticles showed a dose-dependent increase in rifampin exposure, as quantified by area under the concentration–time curve (AUC), with a statistically significant difference between the 5% and 20% β-C-P nanoparticle groups (*P* < 0.05; Fig. 3B). These results reinforce the superior pulmonary retention and delivery efficiency of the β-C-P formulation. The oral rifampin concentration was chosen based on our unpublished results, where we administered a single dose of 40% β-C-P nanoparticles and noted changes in histology, whereas 20% β-C-P nanoparticles had no appreciable change to histology. As the β-C-P nanoparticles are maximally loaded, the 20% dose was chosen as the highest dose for this multi-dose trial. We noted no abnormal pathology in the lungs of mice dosed at 20% β-C-P nanoparticles for 4 weeks. We matched the daily rifampin oral gavage dose to the 20% β-C-P nanoparticles. This choice resulted in having a reduced rifampin exposure (4.33 mg/kg) in the daily oral dose compared to standard of care (10 mg/kg). To evaluate the potential for drug accumulation, we compared the amount of rifampin in the BAL cell pellets following repeat dosing. Mice receiving a second dose on day 7 exhibited higher rifampin levels at 24 h post-dose (i.e., day 8) compared to those receiving only a single administration on day 1 (Fig. S1). However, by day 14, no differences were observed between groups receiving 20% or 10% β-C-P nanoparticle doses (Fig. 2A). Combining the day 8 results along with Fig. 3A, we conclude that a single administration of β-C-P nanoparticles is required to reach steady state in the BAL cell pellet, and there is no evidence of rifampin accumulation over time in healthy mice. These findings suggest that β-C-P nanoparticles deliver a sustained, yet non-accumulative, dose of rifampin to the lungs. Finally, the amount of rifampin detected in the BAL following both a single administration (short timeline [2–8 h]) (12) and multiple weekly administrations (long timeline [7–28 days]) (Fig. 3) demonstrates a significant improvement in the exposure of rifampin (AUC) to the lung compared to oral rifampin. This significantly increased AUC compared to oral rifampin, in combination with serum concentrations that were below the limit of detection, could enable reduction of the inhaled dose to mitigate dose-limiting side effects, including drug-drug interactions, or, if the well-tolerated 20% β-C-P nanoparticle dose is continued, it could result in better control of lung-localized TB.

### Changes in body weight of mice

**Fig 4:**
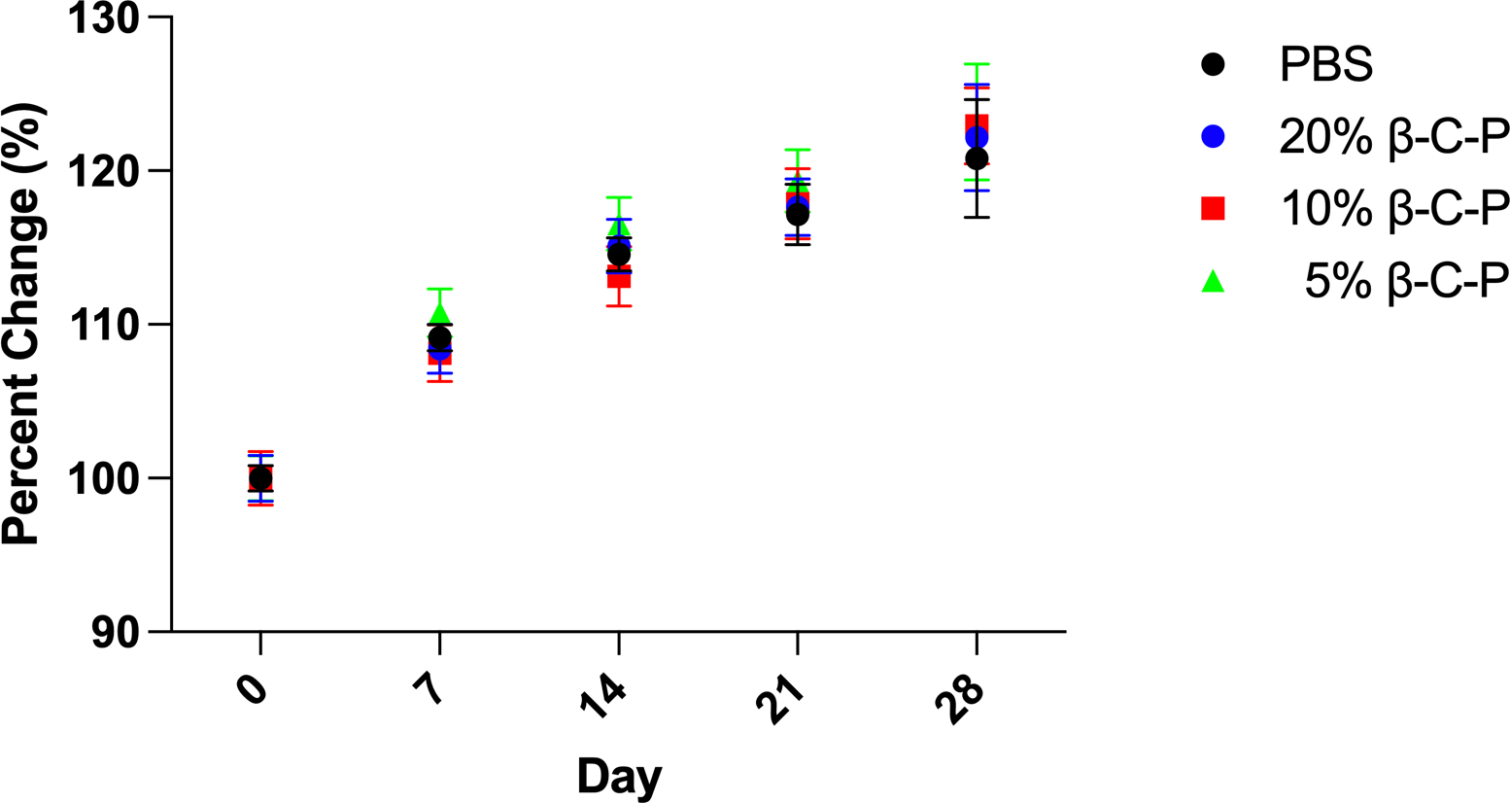
Analysis of body weight changes following repeated inhalation of β-C-P nanoparticles. Mice were weighed weekly on treatment days 0, 7, 14, 21 and on day 28. 20%, 10%, or 5% β-C-P by OPA were compared to PBS control at the respective time points. Statistical analysis was done by a two-way ANOVA followed by a Dunnett’s multiple comparisons test. Data are presented as percent change in body weight. Data shown represent the mean ± SEM (*n* = 4).

CD1 mice were weighed on treatment days (days 0, 7, 14, 21) and at the end of the study (day 28) (Fig. 4). There was no difference in body weight between the β-C-P nanoparticle groups compared to PBS control. These findings demonstrate that repeated OPA-based administrations at 7 day intervals do not produce adverse systemic effects.

### Immune cell populations in the lung following nanoparticle exposure

**Fig 5:**
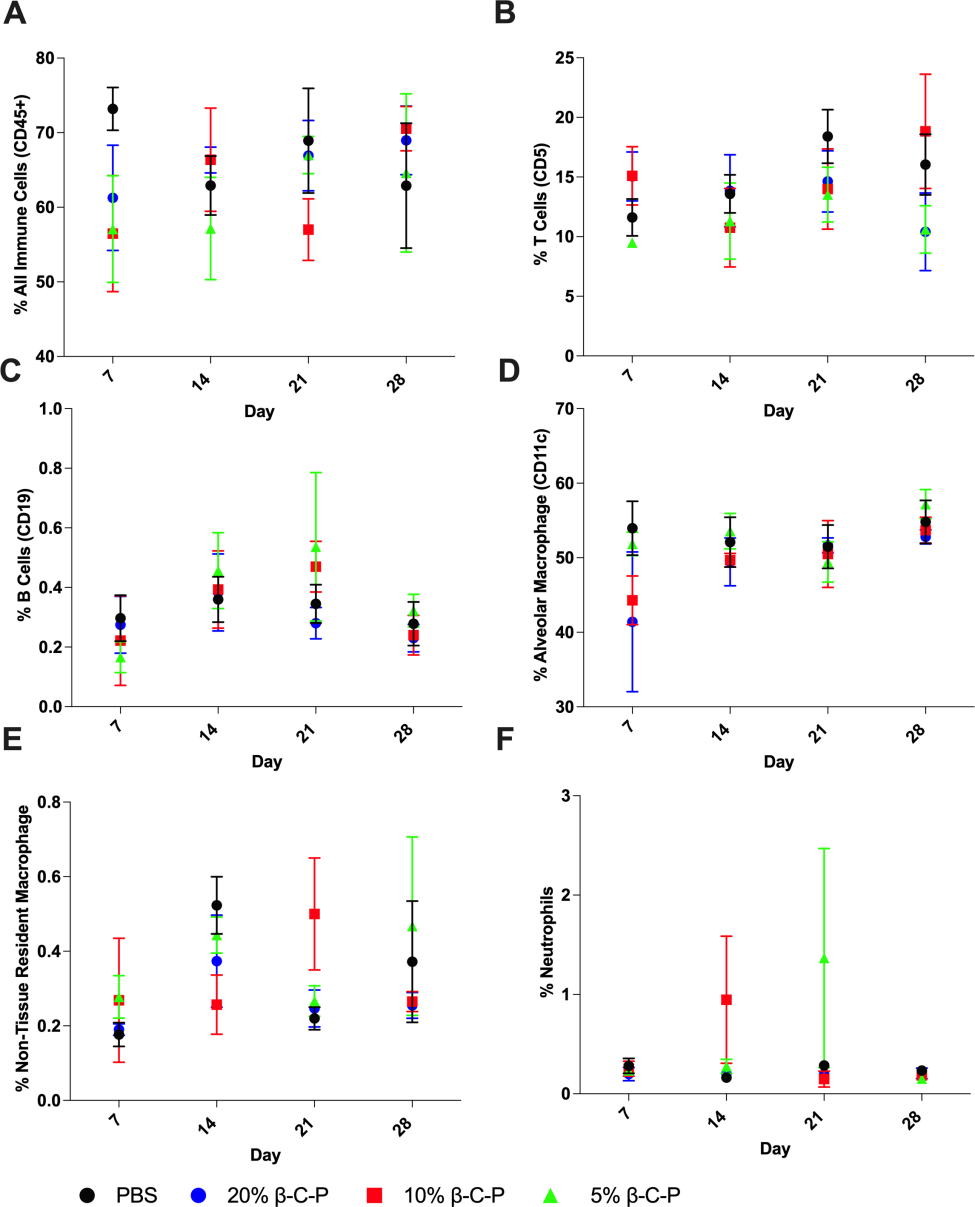
Immune cell populations in the lung following repeated inhalation of β-C-P nanoparticles. Cell populations were analyzed following multiple exposures to 20%, 10%, or 5% β-C-P via OPA every 7 days for 4 weeks. (**A**) All immune cells (CD45), (**B**) T cells (CD5), (**C**) B cells (CD19), (**D**) alveolar macrophages (CD11c, CD11b), (**E**) non-tissue-resident macrophage (CD11b), and (**F**) neutrophils (CD11b, Ly6C). Statistical analysis was performed using a two-way ANOVA followed by Dunnett’s multiple comparisons test. All data are compared to PBS at the respective time points. Data shown represent the mean ± SEM (*n* = 4).

To assess the potential immunological effects of repeated nanoparticle exposure, lung immune cell populations were analyzed 7 days after each dose during a 28 day treatment period (Fig. 5). The cell types analyzed include all immune cells (CD45), T cells (CD5), B cells (CD19), alveolar macrophages (CD11c, CD11b), non-tissue-resident macrophages (CD11b), and neutrophils (CD11b, Ly6C). These markers were selected to provide a broad overview of both innate and adaptive immune responses in the pulmonary environment following nanoparticle exposure. Compared to PBS control at each time point or across time points, there was no significant difference with 20%, 10%, and 5% β-C-P nanoparticles over the 28 day time course of CD45+ immune cells, T cell (CD5+) populations, B cells (CD19+), alveolar macrophage (CD11c+, CD11b+), and non-tissue-resident macrophage (CD11b+) (Fig. 5A through E). These findings indicate that the repeated administration of β-C-P nanoparticles did not induce a general inflammatory response or immune cell infiltration in the lung, did not trigger adaptive immune activation or lymphocyte recruitment to the lung tissue, and did not result in local antigenic stimulation or chronic immune activation in the lung environment. Neutrophils (CD11b+, Ly6C+) (Fig. 5F) followed a similar trend; however, there was a significant difference between the 5% β-C-P nanoparticle group and PBS control at 21 days (after three doses of NPs) (*P* = 0.03), which returned to baseline by day 28, indicating that the response was temporary and self-limiting. However, this increase was not observed in the higher-concentration β-C-P groups (10% or 20%), suggesting that the finding may not represent a consistent or dose-dependent effect. Collectively, these results demonstrate that repeated weekly administration of nanoparticles by the OPA route does not elicit significant immune activation or cellular infiltration in the lung, supporting the biocompatibility of this system for chronic TB therapy. These findings support the immunological safety and biocompatibility of the tested nanoparticle systems for pulmonary delivery of rifampin, which is especially relevant for chronic treatment regimens where minimizing off-target immune responses is critical.

### Cytokine secretion

**Fig 6:**
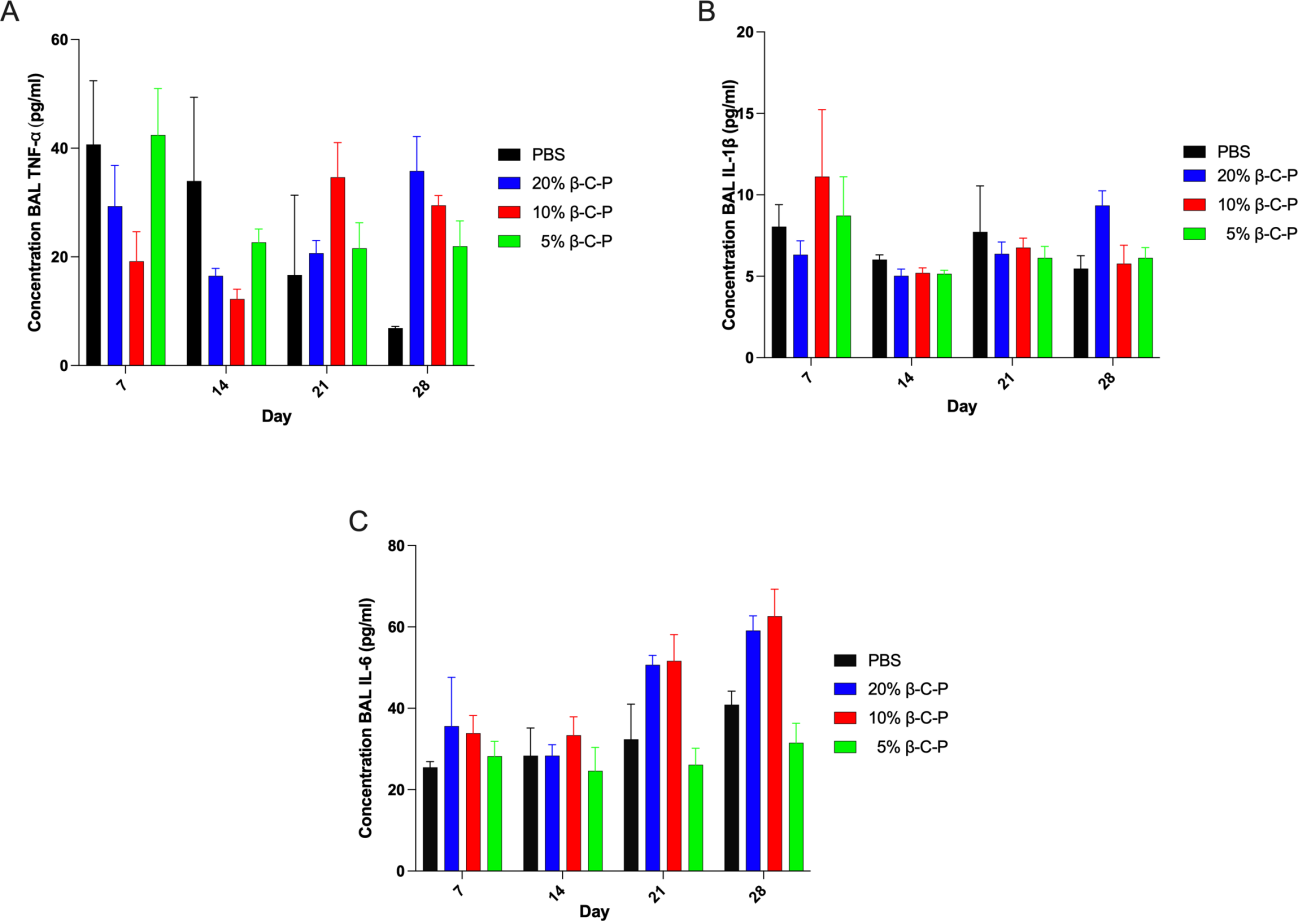
Cytokine analysis in BAL supernatant following repeated inhalation of β-C-P nanoparticles. Cytokine secretion was measured in BAL supernatant at days 7, 14, 21, and 28 following OPA of 20%, 10%, or 5% β-C-P nanoparticles every 7 days for 4 weeks. (**A**) TNF-α, (**B**) IL-1β, and (**C**) IL-6. Statistical analysis was done by a two-way ANOVA with Dunnett’s multiple comparisons test. Data are compared to PBS alone at the respective time points. Data shown represent the mean ± SEM (*n* = 4).

We next evaluated the long-term effects of multiple doses of rifampin-loaded β-C-P nanoparticles on lung inflammation, as measured by cytokine secretion in BAL fluid (Fig. 6). Specifically, we focused on the levels of key pro-inflammatory cytokines, TNF-α, IL-1β, and IL-6, over a time course from 7 to 28 days post-treatment. There was no difference in TNF-α, IL-1β, and IL-6 in the β-C-P nanoparticle treatment groups compared to control (Fig. 6). While the data show no significant changes in TNF-α, IL-1β, or IL-6 levels from 7 to 28 days post-treatment, this absence of detectable cytokine response does not necessarily indicate a lack of immunological activity. In our previous study, we observed measurable cytokine elevations, including TNF-α, IL-1β, and IL-6, within 72 h following treatment (12). This suggests that the cytokine response to these nanoparticle formulations may be acute and transient, peaking early and resolving before the earliest time point examined in the current study.

### Albumin in the lungs

The concentration of albumin in BAL supernatant was measured as an indicator of alveolar epithelial integrity (12) following β-C-P administration by OPA (Fig. S2). Maintaining alveolar epithelial integrity is critical for preserving lung function. At 7 days post-treatment, there was a difference between PBS control and 10% β-C-P nanoparticles; however, there was no difference in any of the other groups. At subsequent time points (14, 21, and 28 days), no significant differences in albumin levels were observed across any of the treatment groups, further supporting the conclusion that repeated dosing with these formulations does not compromise epithelial barrier integrity over time.

### Serum IgG concentrations

The concentration of serum IgG was measured to assess the systemic immune response caused by repeat administration. Compared to PBS control, there was no difference in serum IgG concentration for all treatment groups at each time point and across the duration of the study (Fig. S3). The results indicate the levels of immunoglobulin remain consistent; therefore, a systemic adverse immune reaction is not occurring from repeated treatments.

### Efficacy studies

**Fig 7:**
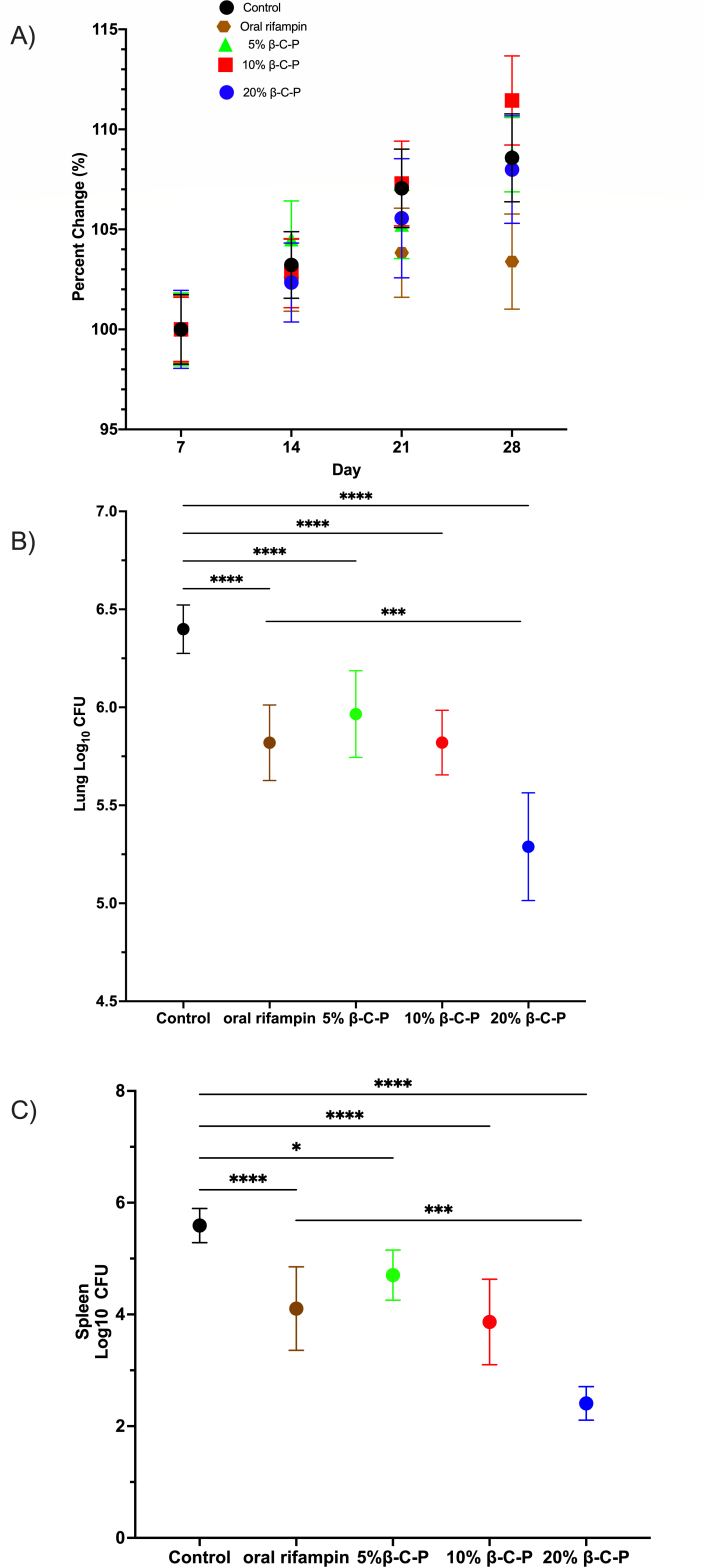
Efficacy studies in a BALB/c TB mouse model following repeated inhalation of β-C-P nanoparticles. Mice were infected with *Mtb*; on day 28 (T0), mice were dosed every 7 days with β-C-P nanoparticles by OPA up to day 21. Oral rifampin was administered by gavage 5 out of 7 days for 4 weeks. Lungs and spleen were harvested at TD28. (**A**) Weekly mouse weights (% change in body weight), starting on T0. (**B**) Lung log_10_ CFU. (**C**) Spleen log_10_ CFU. Statistical analysis was performed by one-way ANOVA followed by Dunnett’s multiple comparisons test. Data are compared to control or oral rifampin. Data shown represent the mean ± SEM (*n* = 7-14). *P ≤ 0.05; *** = *P* ≤ 0.001; **** = *P* ≤ 0.0001. CFU, colony forming units.

Building on these findings in healthy CD1 mice, we next assessed the efficacy of rifampin-loaded β-C-P nanoparticles administered intrapulmonary via OPA in two low-dose *Mtb* mouse models: the BALB/c model (22) and the C3HeB/FeJ Kramnik model (24, 32). In the BALB/c group, nanoparticles were delivered once weekly (T0, T7, T14, T21), while oral rifampin (4.33 mg/kg) was administered by gavage 5 days per week for 4 weeks. On treatment day 28 (T28), lungs were harvested for CFU determination. As shown in Fig. 7A, in the BALB/c model, all groups steadily gained weight throughout the study, indicating tolerability of both nanoparticle and oral rifampin regimens. Behavioral assessments conducted throughout the study revealed no signs of distress, and all mice scored zero on the murine sepsis scale (data not shown) (38). Importantly, daily oral rifampin, 5%, 10%, and 20% β-C-P nanoparticles significantly reduced lung CFU compared to untreated controls. The magnitude of reduction ranged from 0.5 to 1.11 log values, indicating that weekly nanoparticle dosing achieved bactericidal efficacy comparable to daily oral rifampin (Fig. 7B). A significant difference was observed between the 20% β-C-P nanoparticle group and the daily oral rifampin group, indicating that the 20% β-C-P nanoparticles reduced the bacterial lung burden more effectively than daily oral rifampin. Furthermore, spleen CFU (Fig. 7C) was significantly reduced by daily oral rifampin and all β-C-P nanoparticles concentrations, suggesting effective control of systemic bacterial dissemination. Similar to the lung, a statistically significant difference was observed between the 20% β-C-P nanoparticle group and daily oral rifampin. These results align with previous studies reporting enhanced antimicrobial activity of rifampin when encapsulated in nanoparticle systems.

**Fig 8:**
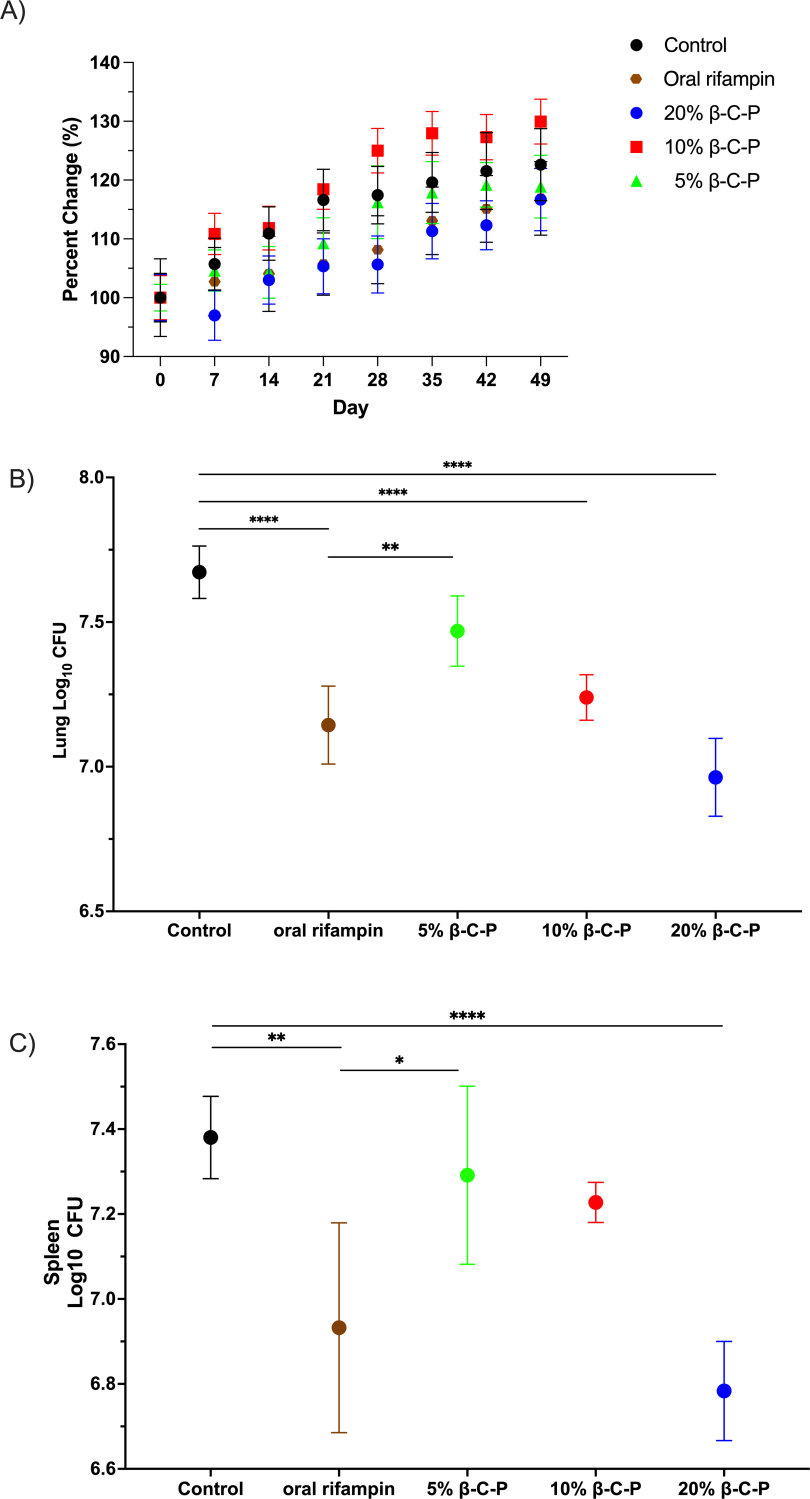
Efficacy studies in a Kramnik *Mtb* mouse model following repeated inhalation of β-C-P nanoparticles. Mice were infected with *Mtb*; on day 42 (T0), mice were dosed every 7 days with β-C-P nanoparticles by OPA for 8 weeks. Oral rifampin was administered by gavage 5 out of 7 days for 8 weeks. Lungs and spleen were harvested at day 98 (TD56). (**A**) Weekly mouse weights (% change in body weight), starting on T0. (**B**) Lung log_10_ CFU (T56). (**C**) Spleen log_10_ CFU (T56). Statistical analysis was performed by one-way ANOVA followed by Dunnett’s multiple comparisons test. Data are compared to control or oral rifampin. Data shown represent the mean ± SEM (*n* = 5–7). *P ≤ 0.05; **P ≤ 0.01; ****P ≤ 0.0001.

As shown in Fig. 8A, in the C3HeB/FeJ Kramnik model, there was no statistical difference in body weight between control groups, daily oral rifampin, and weekly β-C-P nanoparticles, indicating that the C3HeB/FeJ mice tolerated these treatments. Efficacy studies are shown in Fig. 8B. Daily oral rifampin (7.1 CFU ± 0.06), 20% β-C-P nanoparticles (6.9 ± 0.07 CFU), and 10% β-C-P nanoparticles (7.2 CFU ± 0.03) significantly reduced lung CFU compared to untreated controls (7.7 ± 0.04 CFU). Weekly administration of 10% and 20% nanoparticle formulations resulted in bactericidal efficacy comparable to that of daily oral rifampin, as no significant differences were detected between these groups. However, the 5% formulation was less effective, showing significant differences compared to daily oral rifampin. Spleen CFU counts are shown in Fig. 8C. Both daily oral rifampin and weekly 20% β-C-P nanoparticles produced a significant reduction in splenic bacterial load compared to the untreated control, indicating effective control of systemic infection. The reduction in spleen CFU suggests that daily oral rifampin and 20% β-C-P nanoparticles not only limited pulmonary bacterial growth but also helped prevent dissemination of bacteria to extrapulmonary organs. In contrast, lower-dose nanoparticle formulations (5% and 10%) did not achieve significant spleen bacterial reduction, highlighting a dose-dependent systemic efficacy of the β-C-P nanoparticle delivery system.

## DISCUSSION

Several groups have explored the use of PLGA-based drug delivery for TB (15, 43, 44). Since the early 2000s, PLGA has been recognized as a promising vehicle for controlled drug release of rifampin (45, 46) and is already used in FDA-approved long-acting (LA) formulations (47). Inhalable PLGA nanoparticles co-encapsulating isoniazid, pyrazinamide, and rifampin have demonstrated increased plasma AUC and reduced pulmonary bacterial burden in guinea pigs (15), although these benefits were not superior to daily free-drug treatment. More recently, oral administration of ^99^Tc-labeled rifampin-loaded PLGA nanoparticles demonstrated favorable biodistribution and safety profiles in humans (48), supporting the translational potential of this platform. Other strategies have incorporated targeting ligands like mannose (49) and formulations optimized for inhalation (48, 50); however, many of these lack immunomodulatory features.

Surface functionalization with β-glucan and chitosan not only enhances uptake by alveolar macrophages but also provides immunomodulatory benefits (11–13, 43), potentially offering advantages over conventional ligands such as mannose or antibody-conjugated systems. β-Glucan functionalization has shown promise in enhancing intracellular drug retention and macrophage targeting (11–13). In our study, β-C-P nanoparticle administration did not increase immune cell infiltration in the lung (Fig. 4), suggesting that the absence of rifampin accumulation is likely due to the release kinetics and biodegradation profile of PLGA rather than cellular recruitment. The clinical relevance of delivering well-tolerated β-C-P nanoparticles via the pulmonary route lies in their potential to improve TB treatment outcomes. Rifampin, a front-line anti-TB drug, has a short half-life (~3 h), necessitating its administration multiple times per day. The β-C-P nanoparticle formulation sustains therapeutic drug levels in the lung for at least 1 week (12), enabling once-weekly administration. This reduced dosing frequency could significantly improve patient compliance, which is critical for preventing the emergence of MDR-TB. Reduced systemic exposure, as evidenced by undetectable serum rifampin levels, may also mitigate dose-limiting side effects and drug-drug interactions. Our previous pharmacokinetic data support the controlled, lung-localized delivery provided by β-C-P nanoparticles. A single OPA dose led to an ~65-fold increase in pulmonary rifampin AUC (2–8 h) compared to oral gavage, with drug levels detectable for up to 10 days (12). In contrast, in the oral rifampin group, rifampin was undetectable in recovered alveolar cells at 24 h. After four weekly doses, rifampin from β-C-P nanoparticles remained detectable in BAL fluid from days 7–28, while rifampin was not detectable in the oral rifampin group at study end. There was no measurable serum rifampin, no drug accumulation in lung BAL, and no increase in alveolar macrophage numbers, indicating controlled release rather than immune clearance. Building on our previous short-duration study, we extended our evaluation to a 4 week dosing regimen. This longer-term study aimed to assess the pharmacokinetics, safety, and therapeutic efficacy of the formulation in a murine model. To quantify drug delivery to the target site, we measured rifampin levels in the BAL cell pellet at weekly intervals. Notably, rifampin concentrations remained below the limit of detection in animals that received daily rifampin via oral gavage, indicating limited pulmonary delivery by this route. In contrast, mice treated with β-C-P nanoparticles exhibited a clear dose-dependent increase in intracellular rifampin exposure, as measured by the AUC. Importantly, the difference in AUC between the 5% and 20% β-C-P groups reached statistical significance, further supporting the formulation’s efficiency in enhancing local drug retention and cellular uptake. Flow cytometry revealed no significant changes in lung immune cell populations (CD45+, CD5+, CD19+, CD11b+, Ly6C+), aside from a transient increase in neutrophils in the 5% β-C-P group at day 21 (*P* = 0.03), which resolved by day 28. BAL cytokine levels (TNF-α, IL-1β, and IL-6) remained unchanged, and stable albumin levels indicated preserved alveolar epithelial integrity. Serum IgG levels remained consistent, indicating no systemic immune activation or humoral response against the nanoparticle. These results support the immunological safety of repeated nanoparticle administration. Weekly OPA dosing did not impact weight gain or sepsis scores in mice (38, 51), further confirming the absence of systemic toxicity. While β-glucan is known to be immunostimulatory, its presence on the nanoparticle surface did not trigger excessive or sustained cytokine production. This balance between enhanced cellular uptake and limited immunogenicity is a favorable outcome for pulmonary drug delivery systems. Importantly, there were no signs of cytokine storm or chronic inflammation, key concerns for long-term therapies.

Our current study investigates efficacy in two TB-infected mouse models. In a low-dose *Mtb* BALB/c model, weekly OPA administration of 5%, 10%, and 20% β-C-P nanoparticles significantly reduced lung CFU by 0.5–1.11 log_10_, comparable to daily oral rifampin (4.3 mg/kg). Similarly, in the C3HeB/FeJ mouse model, which develops necrotic granulomas resembling human TB, weekly β-C-P nanoparticle treatment (10% and 20%) reduced lung CFU to levels comparable to daily oral rifampin. These findings suggest that weekly nanoparticle-based therapy can match the efficacy of conventional daily dosing while reducing systemic exposure and potentially improving compliance. This study provides the first report of rifampin-loaded β-C-P nanoparticles evaluated in both the BALB/c and C3HeB/FeJ TB models. We have previously characterized their short-duration pharmacokinetics, immunomodulatory effects, and macrophage-targeting ability (12–14). The current findings confirm their safety, tolerability, and therapeutic efficacy following weekly OPA administration for 28 days or 56 days. Lung integrity was preserved, systemic exposure was negligible, and bacterial burden was reduced to levels comparable to daily oral rifampin administration, positioning this platform as a promising candidate for LA, inhalable TB therapy. This study also highlights that therapies that appear effective in BALB/c mice may fail to demonstrate efficacy in Kramnik mice (24, 33, 34). The 5%, 10%, and 20% β-C-P nanoparticles were effective in the BALB/c model, whereas only the 10% and 20% formulations were effective in the Kramnik model, demonstrating the importance of efficacy testing in both models.

LA medication therapies have improved adherence compared to daily oral therapy, leading to better clinical outcomes (52). Lack of adherence is one of the most critical factors contributing to MDR-TB (53). Subtherapeutic drug levels from missed or irregular doses allow antibiotic concentrations to fall below the minimum inhibitory concentration (MIC), creating fluctuating drug levels that enable bacteria to survive and multiply. Low or inconsistent concentrations exert selective pressure, favoring partially resistant strains and can promote resistance mechanisms such as increased mutation rates, gene transfer, and activation of efflux pumps (54, 55). TB treatment is long, typically lasting 6 to 9 months, and many patients start feeling better within a few weeks, which can lead them to stop taking their medication prematurely. Some patients may also adjust their doses on their own to avoid side effects, resulting in subtherapeutic levels that promote the survival of resistant strains. By reducing dosing frequency, LA therapies make it more likely that patients will complete treatment, improving effectiveness and reducing the risk of resistance (52). A recent study by Balu et al. developed injectable in-situ gels using poloxamer 407, carbopol 940, and hydroxypropyl methylcellulose to deliver rifampicin and isoniazid (56). The optimized formulation demonstrated ideal gelation at 26°C, high drug content, and stable physicochemical properties. *In vitro* studies showed controlled release of rifampicin and isoniazid for up to 10 and 6 days, respectively, with no cytotoxicity observed in cell models. Designed for intramuscular administration, this system could reduce dosing frequency and improve patient adherence in chronic TB therapy, although it has yet to undergo *in vivo* evaluation. Building on similar goals of sustained TB drug delivery, Kim et al. employed *in situ* forming implant technology to fine-tune rifabutin release rates (57). The inclusion of amphiphilic additives such as Kolliphor HS 15, TPGS, and Tween 80 markedly enhanced rifabutin solubility and loading capacity, minimized the initial burst release, and extended drug release both *in vitro* and *in vivo*. In animal studies, the optimized high-load rifabutin formulation (RFB14KH) maintained plasma drug concentrations more than ten-fold above the MIC for at least 4 weeks and above the MIC for up to 16 weeks, with substantial drug accumulation in lung and spleen tissues. Together, these studies highlight complementary approaches to achieving LA injectable therapies that could transform TB treatment by improving adherence and reducing dosing frequency.

Inhalable PLGA particles co-encapsulating isoniazid, pyrazinamide, and rifampin have demonstrated increased plasma AUC and reduced pulmonary bacterial burden in guinea pigs (15), although these benefits were not superior to daily oral drug treatment. Most importantly, this study demonstrates the ability to co-encapsulate first-line TB drugs. The co-encapsulation of rifampin, isoniazid, and pyrazinamide within a single β-C-P nanoparticle system presents several potential advantages but also notable challenges. Although such a formulation could replicate the synergistic effects of standard first-line TB therapy while enhancing intracellular delivery and reducing the risk of resistance, its development is hindered by significant physicochemical and formulation constraints (15). Rifampin is relatively lipophilic, whereas isoniazid and pyrazinamide are hydrophilic, making it difficult to efficiently encapsulate all three agents (58). Moreover, chemical incompatibilities exist between rifampin and isoniazid, as these drugs can form inactive hydrazone derivatives under acidic conditions, while each compound also exhibits different pH stability profiles (44). Achieving synchronized release kinetics further complicates formulation design, since variations in solubility and molecular weight can lead to unequal drug release and subtherapeutic exposure. Therefore, while combining rifampin, isoniazid, and pyrazinamide in a single nanoparticle system remains an attractive strategy for improving therapeutic efficacy and compliance, overcoming these formulation and translational barriers is essential for successful clinical application. Future studies will focus on developing a β-C-P nanoparticle system that can combine rifampin, isoniazid, and pyrazinamide in a single nanoparticle system.

### Conclusion

These findings support the feasibility of nanoparticle-based pulmonary delivery of rifampin as a LA, biocompatible, and immunologically safe alternative to conventional daily oral TB therapy. Weekly administration of β-C-P nanoparticles maintained therapeutic lung concentrations, reduced bacterial burden, and avoided systemic toxicity or immune activation. This approach may simplify TB treatment, improve patient adherence, and reduce side effects, key goals in addressing both drug-sensitive and drug-resistant TB. Continued pre-clinical and translational research is warranted to further optimize this platform and evaluate its performance.

## SUPPLEMENTAL MATERIAL

Supplemental material aac.01480-25-s0001.docx Fig. S1 to S3.

ASM does not own the copyrights to Supplemental Material that may be linked to, or accessed through, an article. The authors have granted ASM a non-exclusive, world-wide license to publish the Supplemental Material files. Please contact the corresponding author directly for reuse.
