# Supplementary material for: Repeated pulmonary dosing of β-glucan-chitosan-PLGA nanoparticles controls Mycobacterium tuberculosis in mice
Source: Antimicrob Agents Chemother. 2026 Jan 14;70(2):e01480-25. doi: 10.1128/aac.01480-25 (PMC12888923; doi:10.1128/aac.01480-25)
Supplement: Supplemental material — Fig. S1 to S3. [file aac.01480-25-s0001.docx]

*Supplemental figures*

**Figure S1. Analysis of rifampin in the BAL cell pellet following repeated inhalation of β-C-P nanoparticles.** Rif recovered Day 8 of treatment either single dose (Day 8 20% - 20% β-C-P nanoparticle dose on Day 1, BAL sample collected on Day 8) or double dose (Day 8 20% 10% - 20% β-C-P nanoparticle dose on Day 1, 10% β-C-P nanoparticle dose on Day 7, BAL sample collected on Day 8) of β-C-P nanoparticles. A one-way ANOVA with a Tukey’s multiple comparisons test had no significant results. Data shown represent the Mean ± SEM; (n = 4).

**Figure S2. Albumin concentration in the BAL supernatant following repeated inhalation of β-C-P nanoparticles.** Albumin secretion was measured in BAL supernatant at Day 7, 14, 21, and 28 following OPA administration of 20%, 10%, or 5% β-C-P nanoparticles (weekly administration). Statistical analysis was done using a two-way ANOVA followed by Dunnett’s multiple comparisons. All data are compared to PBS at respective time points. Data shown represent the Mean ± SEM; (n = 4).

**Figure S3. IgG in the serum supernatant following repeated inhalation of β-C-P nanoparticles.** IgG concentrations were measured in serum at Day 7, 14, 21, and 28 following OPA administration of 20%, 10%, or 5% β-C-P nanoparticles (weekly administration). Statistical analysis was done using a two-way ANOVA followed by Dunnett’s multiple comparisons. All data are compared to PBS at respective time points. Data shown represent the Mean ± SEM; (n = 4).
